# First-principles calculations of the electronic and possible topological properties of confirmed two-dimensional high *T*_c_ superconductors Nb_2_CoS_4_, Nb_2_CuS_4_, and PtN_2_

**DOI:** 10.1039/d5ra06727h

**Published:** 2025-10-20

**Authors:** Haolin Lu, Sophia Ren, Xuan Luo

**Affiliations:** a National Graphene Research and Development Center Springfield Virginia 22151 USA cx3301234@gmail.com

## Abstract

Recently, next generation two-dimensional high-*T*_c_ superconductors have attracted intense interest. Motivated by this development, we had great interests to explore whether these high-*T*_c_ superconducting monolayers has topological properties. Here, a systematic investigation of three high-temperature, two-dimensional superconductors Nb_2_CoS_4_, Nb_2_CuS_4_, and PtN_2_ was conducted to examine their electronic band structures. Their band structures without SOC displayed Dirac-like crossings near the Fermi level. Upon including SOC, we found virtual evidence for locally gapped Dirac-like crossings in hexagonal PtN_2_ and SOC-resolved nodal-ring features in the two niobium compounds. These phenomena indicated possible topologically nontrivial phases, suggesting that these high-*T*_c_ superconductors are possible candidates for the ongoing search for topological superconductivity.

## Abstract 2

Electronic band structure calculations with SOC were conducted to ascertain possible topological properties of 2D high-*T*_c_ SCs.

## Graphical abstract

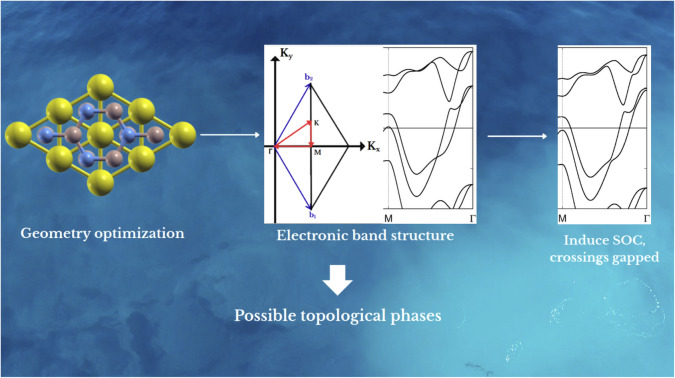

## I. Introduction

High-temperature superconductors (HTSs), which remain superconductive above the boiling point of liquid nitrogen (−195.8 °C), have always been the central interest among researchers since the discovery of superconductivity by Heike Kamerlingh Onnes in 1911.^1^ Then, up to 1975, over 2000 superconducting materials were found, and although the critical temperature (*T*_c_) gradually rose, reaching 22.3 K with the discovery of Nb_3_Ge in 1973,^2^ the progress stalled until 1986, when the landmark in the field – cuprate HTSs – were discovered by Bednorz and Müller.^3^ Their findings marked a pivotal moment in the field, followed by intense research into iron-based superconductors.^4^ More recently, attention has shifted toward layered HTSs, due to their tunable electronic properties, and mechanical flexibility.^5^ The isolation of monolayer NbSe_2_ in 2015 confirmed that superconductivity can truly persist in the 2D limit^6^ led to a rapid expansion in the catalog of 2D superconductors, including monolayers of MoS_2_,^7^ FeSe,^8^ and more.^9^ In addition, a recent high-throughput workflow was used to screen over 1000 2D materials in the JARVIS-DFT database.^10^ This investigation identified 34 dynamically stable monolayer superconductors with *T*_c_ > 5 K. Among these, PtN_2_, Nb_2_CuS_4_, and Nb_2_CoS_4_ emerged as promising platforms, each with *T*_c_ > 8 K, and a remarkable *T*_c_ ≈ 11.9 K for PtN_2_.^11^ The identification of PtN_2_ alongside Nb_2_CuS_4_ and Nb_2_CoS_4_, three metallic compounds, provides an exciting opportunity to explore the potential nontrivial band topology in those high *T*_c_ 2D superconductors.

In a 2D superconductor, it is very straightforward to localize and braid Majorana zero modes. One usually adiabatically exchanges vortex-bound Majorana modes to implement non-abelian unitary operations—the basic prerequisite of topological quantum gates.^12^ In reality, two-dimensional systems have shown great potential in hosting topologically nontrivial phases, as seen in the recent breakthroughs.^13–15^ For example, a 2020 experiment demonstrated a topological superconducting state in a van der Waals heterostructure incorporating a monolayer ferromagnet (CrBr_3_) on a superconducting substrate.^16^ Simultaneously, theoretical studies have identified high-*T*_c_ 2D superconductors with nontrivial band topology.^17^ Few years later, a monolayer boron–carbon compound Mg_2_B_4_C_2_ (which is nonmagnetic and superconducting) was predicted to be topologically nontrivial based on the Fu-Kane parity criterion by utilizing its inversion and time-reversal symmetries (TRS).^10^

Motivated by these developments, we investigated the electronic properties of mono-layer PtN_2_, Nb_2_CuS_4_, and Nb_2_CoS_4_ upon applying SOC by conducting first-pinciples calculations. Starting from these electron–phonon (EPC) based results and taking advantage of the broader JARVIS database,^18^ a detailed analysis on spin–orbit driven phenomena was performed. We compute the electronic band structures with and without SOC. The projected densities of states (PDOS) were then analyzed to confirm metallicity, and corroborate findings from the previous analysis. Our key objective is to test whether conventional EPC-driven superconductivity can be engineered by SOC's effects on metallic monolayers with heavy atoms(Pt and Nb) to potentially exhibit a nontrivial band topology.

In Section II, we detailed our methods to perform first-principle calculations. We present and analyze our results on the spin–orbit driven effects examination of PtN_2_, Nb_2_CoS_4_, and Nb_2_CuS_4_ in Section III. Finally, our conclusion and future work are found in Section IV.

## II. Methodology

### A. Computational details

The current research conducted first principles calculations based on Density Functional Theory (DFT)^19,20^ implemented in ABINIT package.^21,22^ The generalized gradient approximation (GGA) with the Perdew–Burke–Ernzerhof (PBE) functional^23^ were chosen for exchange-correlation functionals. All calculations employed projector augmented wave (PAW) pseudopotentials,^24–26^ which were generated using the AtomPAW code.^27^ Detailed information about the valence configurations and radius cutoff for each element is provided in Table 1.

**Table 1:** Electron configurations and radial cutoffs used for generating PAW pseudopotentials

Element	Atomic number	Electron configuration	Radius cutoff (bohr)
Nb	41	[Ar 3d10] 4s^2^4p^6^5s^1^4d^4^	2.21
Co	27	[Ne] 3s^2^3p^6^4s^1^3d^8^	2.1
S	16	[Ne] 3s^2^3p^4^	1.9
Cu	29	[Ne] 3s^2^3p^6^4s^1^3d^1^O	2.0
Pt	78	[Xe 4f14] 6s^1^5d^9^	2.5
N	7	[He] 2s^2^2p^3^	1.2

### B. Total energy convergence and ground-state calculations

Self-consistent field (SCF) iterations were converged when the total-energy tolerance criteria of 1.0 × 10^−10^ hartree (Ha) was reached in two successive iterations. The total energy difference between two consecutive datasets must fall under 1.0 × 10 Ha twice for plane-wave kinetic-energy cutoffs (*E*_cut_), Brillouin-zone (BZ) *k*-point meshes, and vacuum thickness for slab geometries to converge. They were independently converged for each material; the resulting parameters were then used consistently in structural relaxations and in band-structure calculations with and without spin–orbit coupling (SOC).^28,29^ To improve convergence and smooth out DOS for 2D PtN_2_ and Nb-chalcogenides, we used a converged broadening factor *σ* of 0.012 and 0.005 Ha for the Gaussian smearing^30^ described by the following equation.1
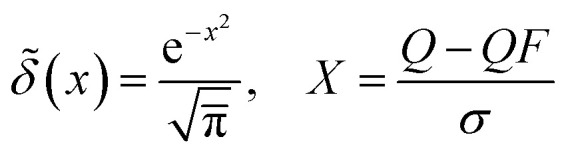


### C. Geometry optimization

Structural relaxations were carried out within the Born–Oppenheimer approximation using the Broyden–Fletcher–Goldfarb–Shanno (BFGS) quasi-Newton algorithm.^31–33^ At each ionic step, the Kohn–Sham equations were solved self-consistently. Hellmann–Feynman forces, *F*_I_ = −*∂E*/*∂R*_I_, were used to update atomic positions.^34^ Relaxations were considered converged when the maximum force component fell below 1.0 × 10^−5^ hartree bohr^−1^. For crystal cell optimizations, the stress tensor was included, and an homogeneous dilation of the components of three lattice parameters is allowed. The relaxed vacuum space Parameters of relaxed structures are shown in Table 2, they were then used for all electronic analyses. The relaxed atomic structures of PtN_2_, Nb_2_CuS_4_, and Nb_2_CoS_4_ are shown in Fig. 2.

**Table 2:** Experimental *vs.* our calculated lattice parameters for each 2D monolayer material

Material	Experimental (Å)^10,11^	Current result (Å)	Error%
PtN_2_	3.2324	3.2684	1.114
Nb_2_CuS_4_	3.6105	3.6326	0.612
Nb_2_CoS_4_	3.3182	3.3254	0.217

### D. Electronic band structure methodology

**Fig. 1:**
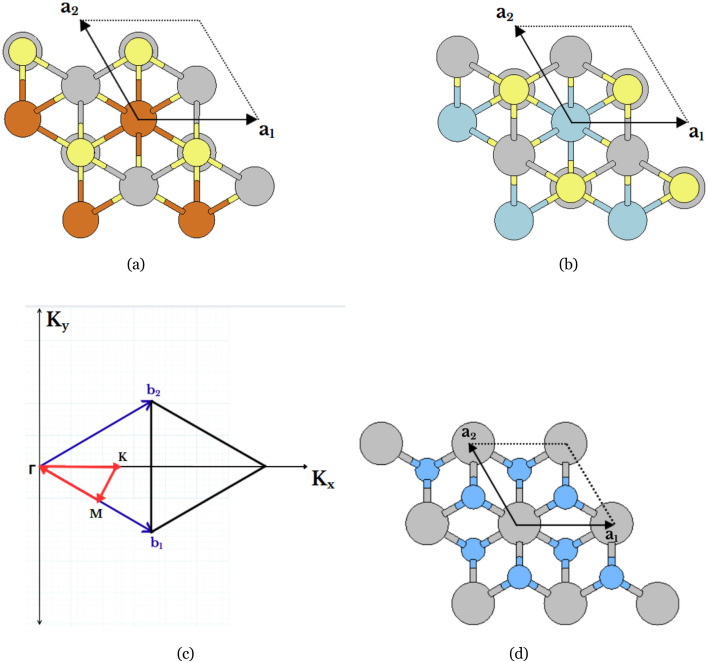
(a)–(c) Top views of atomic structures of Nb_2_CuS_4_, PtN_2_, and Nb_2_CoS_4_. *a*_1_, *a*_2_ represent the lattice vectors of the primitive cell. (d) The red line denotes the high-symmetry *k*-path used for electronic-structure calculations. *b*_1_, *b*_2_ represent lattice vectors in reciprocal space.

Electronic band structures were computed along high-symmetry points of the 2D Brillouin zone using the converged SCF charge density. The Brillouin zone is sampled with a *k* mesh of size 12 × 12 and 8 × 8 for PtN_2_, and two Niobium compounds, respectfully. A vacuum layer of 10, 17, 18 Å is used to avoid residual interactions between adjacent layers. We calculated band structures with and without SOC for each compound. To corroborate electronic properties near *E*_F_ analyzed from band structure, we computed projected density of states (PDOS). In this approach, the PDOS on specified atom and orbital is obtained by projecting Kohn–Sham states *ψ*_*nk*_ onto the corresponding PAW partial waves inside the augmentation spheres and integrating over the BZ. We used the dense *k*-meshes and small electronic broadenings to ensure smooth and well-resolved PDOS. In systems containing d-orbital electrons, a proper *U* value calculation is performed to correct for the underestimation of electron correlation. We considered including a +*U* correction due to the presence of heavy elements such as niobium and platinum. Nonetheless, from our DOS diagram of Pt_2_, Nb_2_CuS_4_, and Nb_2_CoS_4_, we observed that the d-orbitals contribute few states around the Fermi level, so a +*U* would not affect our results critically. For this reason, we did not make use of +*U* in our calculations (Fig. 1).

**Fig. 2:**
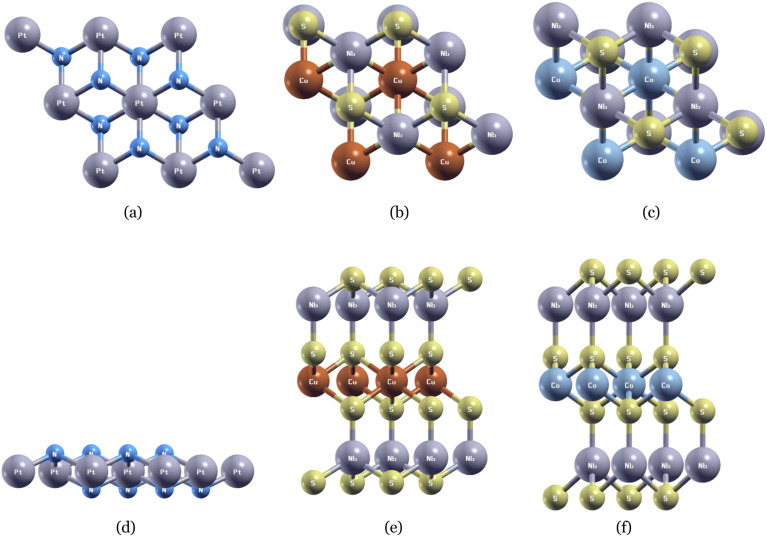
(a–c) Top and (d–f) side views of atomic structure for 2 × 2 supercell of PtN_2_, Nb_2_CuS_4_, and Nb_2_CoS_4_.

## III. Results and discussion

### A. PtN_2_

**Fig. 3:**
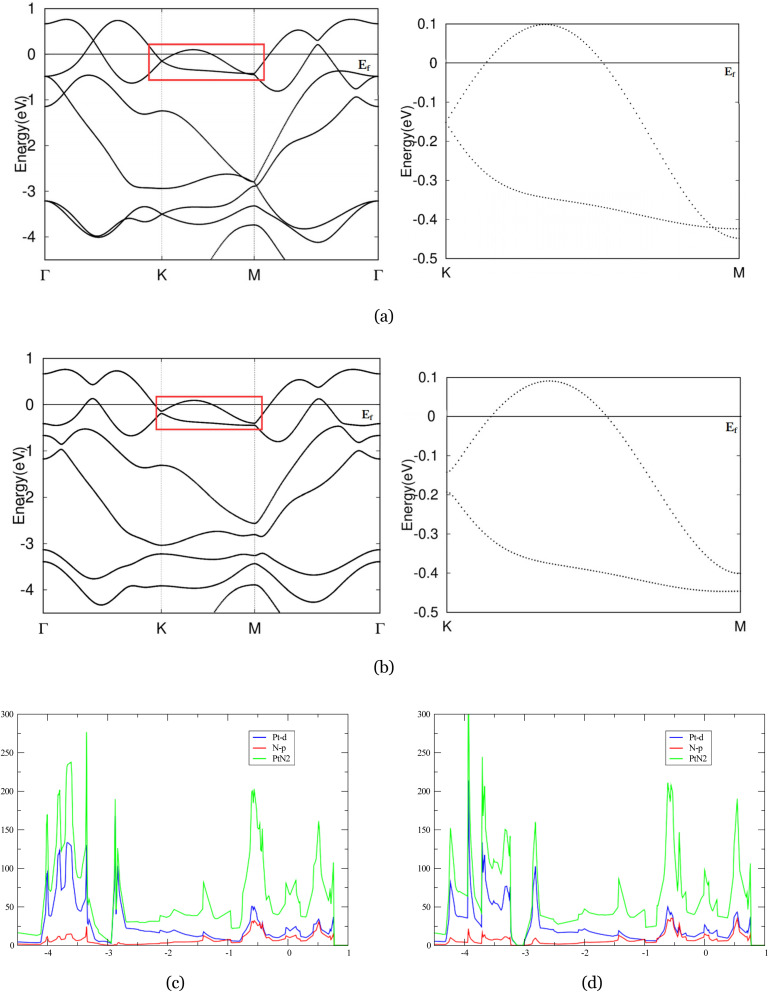
(a) and (b) The band structures of PtN_2_ without and with SOC. Red box in (a) and (b) represent the closed/gapped Dirac-like crossings. (c) and (d) Density of states (DOS) of PtN_2_ without and with SOC.

Comparing the band calculation of PtN_2_ along *Γ*–*K*–*M*–*Γ* without SOC (Fig. 3(a)) and with SOC (Fig. 3(b)), we find virtual evidence for gapped Dirac-like crossings, within our initial prediction that PtN_2_ (heavy Pt) will lose its loops' symmetry protection and open gaps under SOC,^35^ which are evident in previous studies.^36^ Along *Γ* → *K*, SOC preserves the Kramers degeneracy^37^ at *Γ* but the two branches split with parabolic dispersion further along the route. On that region, a notable ∼0.3 eV gap is observed, corroborating a robust SOC effect between *Γ* → *K*. Similarly, its effects push the conduction branch up and the valence branch down in the boxed region, mildly reshaping the curvature (slightly flatter top branch near mid *K* → *M*). Our result compares favorably with previous works investigating topology in 2D MoO,^38^ Nb_2_O_3_,^39^ and RuClBr.^40^ Fittingly, in the presence of SOC, Weyl points observed across the panel are gapped. Our band calculation produces that the SOC has an effect strong enough to indicate a potential metal–insulator transition. Though the full left panel still exhibits other crossings around Fermi level in the Brillouin zone, and a confirmed topological nontrivial phase is doubtful, the gap denoted in red box shows great potentials for the exhibition of nontrivial winding.^41,42^ To make clear of topological properties, in future, one can apply appropriate perturbations/tuning to induce the entire 2D Brillouin zone gapped, and if TRS remains intact, the system can undergo a metal-to-SOC-insulator transition, evident in previous studies,^43,44^ thereby further confirming the doubted topological nontrivial phase.

The DOS diagram shows that PtN_2_ is clearly metallic. The weight at *E*_F_ is dominated by Pt-d states. N-p contributions are more modest and appear mainly between ∼−1.4 to 0.8 eV, where they hybridize with the stronger Pt-d orbital. A prominent Pt-d manifold is also observed between ∼−4.1 and −2.8 eV, consistent with our expectation that PtN_2_'s metallic properties arise from the transition metal atom's d-orbital, and earlier study.^45^ When SOC is included, the system remains metallic: an abundance of DOS is observed at *E*_F_, corroborating the band-structure result that only a local gap opens (along *K* → *M*) rather than a global insulating gap. In the vicinity of *E*_F_, the Pt-d intensity is only mildly smoothed (no full depletion of the peaks). This corresponds to the degeneracies lifted by SOC effects we observed in the band structure diagram. SOC primarily reorganizes the Pt-d orbital, that is, Pt-d peaks are shifted and split slightly. In particular, sharp structures around −3.8 to −3.0 eV transition into smoother shoulders, and the broad feature near −1 eV becomes wider and slightly lower in amplitude. Notably, the N-p orbital track these changes, confirming that the N-p hybridization persists while SOC mainly influences the Pt-d states.

### B. Nb_2_CuS_4_

**Fig. 4:**
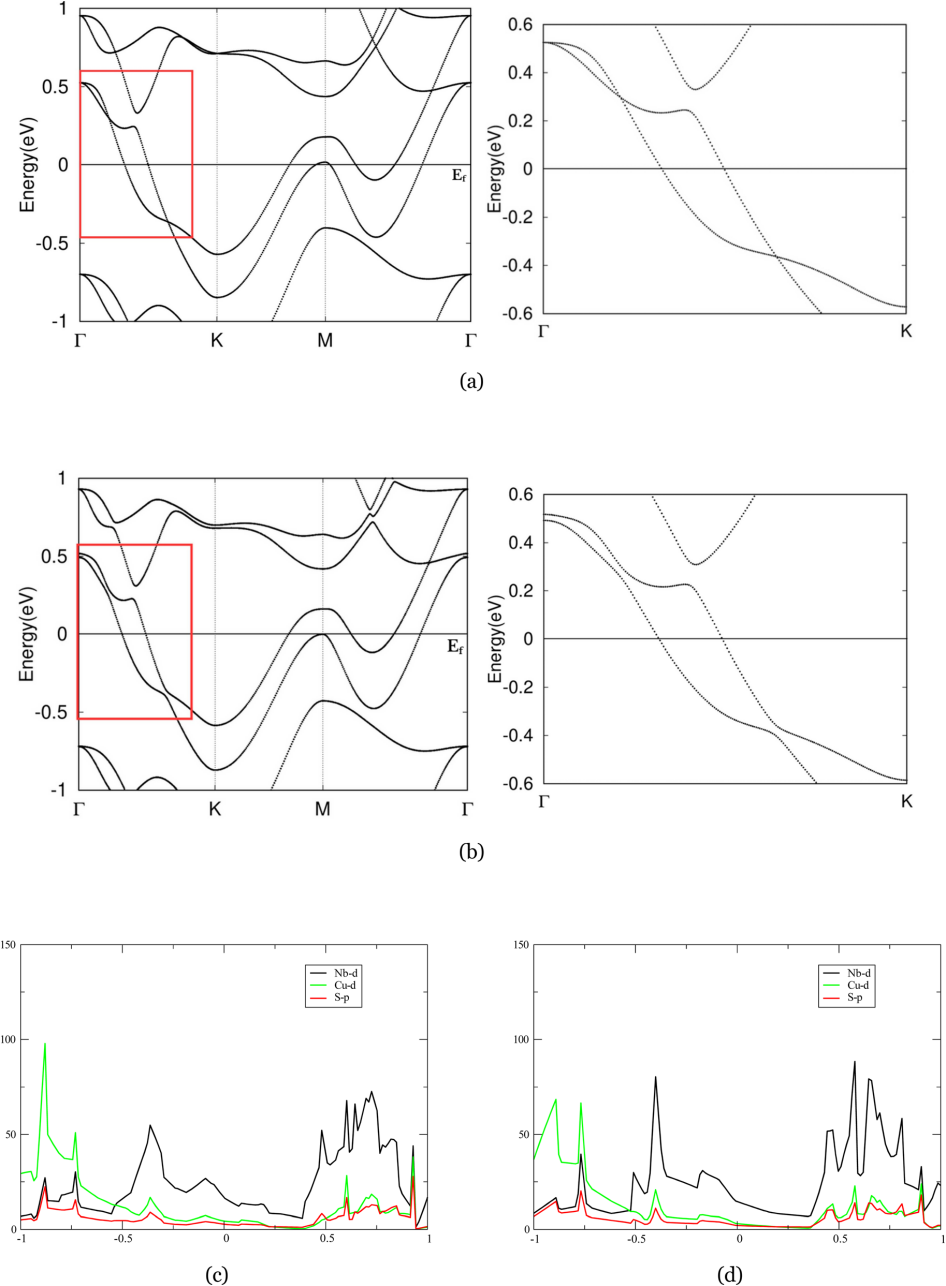
(a) and (b) The band structures of Nb_2_CuS_4_ without and with SOC, the red box in (a) and (b) denoted nodal rings between *Γ* and *K*, visible in magnified *K*-path. (c) and (d) Projected density of states (PDOS) of Nb_2_CuS_4_ without and with SOC.

Fig. 4(a) presents the band structure of monolayer Nb_2_CuS_4_ in the absence of SOC. Our results reproduce the general features reported in previous studies.^46,47^ The conduction and valence bands approach each other along the *Γ*–*K* direction in a curved, parabolic-like fashion, lying above *E*_F_ in the energy window of 0–0.5 eV. Including SOC in band structure calculation gapped band crossings near Fermi level, especially visible along the *K*–*Γ* direction, which is expected since both niobium and copper produce relatively strong SOC. Particularly, we observe avoided crossings or nodal-like features, highlighted in the red box. These features are indicative of quasi-nodal lines that open small gaps once SOC is included.^48,49^ With the incorporation of SOC, nodal ring features are gapped to realize a semi-insulator phase with a direct gap across the *Γ*–*K* path. This compares favorably with previous study on two dimensional hexagonal metal–oxide lattice, Nb_2_O_3_.^39^ The strong strength of SOC resolved crossing, forming topological insulator (TIs) like features. Although a topologically nontrivial phase is uncertain, in future, one can introduce appropriate perturbations to the system, inducing a finite gap across the whole Brillouin zone to further corroborate the doubted topological properties.

The PDOS diagram of Nb_2_CuS_4_ is in good agreement with its band calculation. Near *E*_F_, the DOS is dominated by Nb-d states hybridized with Cu-d and S-p orbitals, affirming the compound's metallicity. Nb-d state's influence spanned over the region near Fermi, and, notably, led to a broad peak near −0.4 eV exhibiting a van-Hove like feature^50^ with additional weight from Co-d and minor S-p mixture. Upon including SOC (Fig. 4(d)), this structure evolves into a sharper, Nb-d dominated peak, consistent with the lifting of parabolic crossing among bands near −0.4 eV, amplified in the red box. SOC splits and collapses the van-Hove structure into a more localized, sharp feature. In addition, this sharpening implies that moderate hole doping (or gating) might drive this DOS feature toward *E*_F_, enhancing affirmed superconductivity^51,52^ near the Fermi level.

### C. Nb_2_CoS_4_

**Fig. 5:**
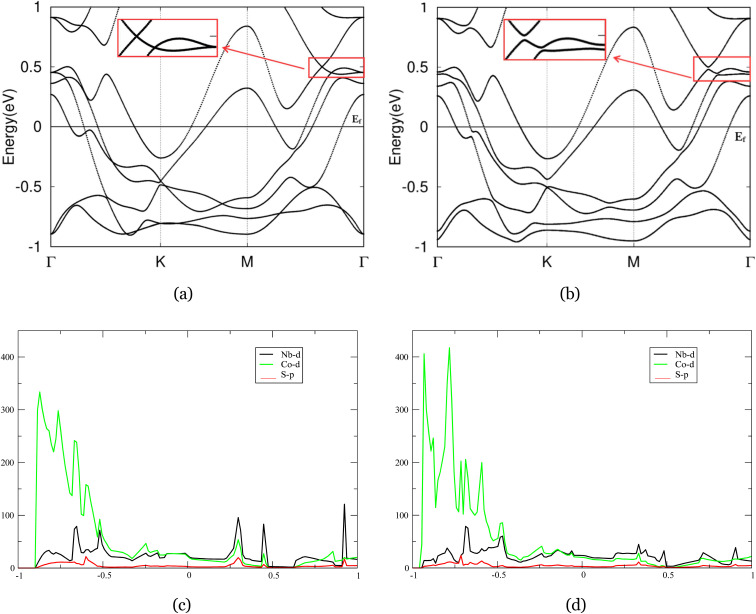
(a) and (b) The band structures of Nb_2_CoS_4_ without and with SOC. The nodal ring along *M*-/gamma path was denoted in the red box, magnified on side. (c) and (d) Projected density of states (PDOS) of Nb_2_CuS_4_ without and with SOC.

As shown in Fig. 5, crossed branches across the panel are lifted, corroborating our expectation that Nb_2_CoS_4_ will produce similarly strong SOC like Nb_2_CuS_4_. Notably, the band structure of Nb_2_CoS_4_ without spin–orbit coupling (left panel) exhibits several susceptible band degeneracies near the Fermi level along the high-symmetry *M*–*Γ* path (highlighted by the red box). These crossings extend robustly along the symmetry lines, strongly suggesting the presence of nodal-ring features that are stabilized by time-reversal symmetries. Similar to Nb_2_CuS_4_, once spin–orbit coupling is introduced (right panel), the bands are gapped and the crossings evolve into small but finite gaps, an indication of symmetry-breaking by SOC. This is consistent with earlier research.^53,54^ The results of strong SOC effects on Nb_2_CoS_4_ are in good agreement with its counterpart, Nb_2_CuS_4_, and other 2D hexagonal lattice, Nb_2_O_3_.^39^ We observed resolved nodal rings and crossings across the *M*–*Γ* path, rendering TI-like features on the right plane. Notably, we also observe the degeneracy at *K* lifted by the strength of SOC. Those are both indicative of a nontrivial band topology in the system, though whether it is a topologically nontrivial phase is doubtful. However, one can induce an energy gap across the entirety of the 2D Brillouin zone through properly tuning the band structure. In this case, the possible topological properties of this system are further affirmed.

In both panels (Fig. 4(c) and (d)), total DOS at *E*_F_ has quite large magnitude, indicating a metallic nature of Nb_2_CoS_4_ governed by substantial Nb and Co-d weight. This yields a more structured spectrum than in the Nb_2_CuS_4_. Two peaks are observed around 0.2 to −0.5 eV, dominated by Nb-d orbital while the Co-d hybridizes. Both of them are then collapsed by the effects of SOC in right panel, in agreement with gapped band crossings and resolved nodal-ring features we observed in band calculations. Besides, in spin–orbit coupling calculation, the overall PDOS shape remains the same—DOS at *E*_F_ is virtually unchanged, suggesting its metallic property is robust against SOC. That said, the sharp peaks broaden slightly under SOC, notably near −0.5 eV. This behaviour is consistent with our observation that degenerate bands near −0.5 eV splitting into adjacent but gapped branches under SOC. Therefore, our DOS diagram under SOC corroborates the resolved nodal-ring features near 0.5 eV, and perturbs the fine spectrum details near −0.5 eV without altering the metallic character at the Fermi level.

## IV. Conclusion

Through a systematic study based on the first-principles density functional calculations, we investigated the atomic, electronic, and SOC properties of 2D PtN_2_ and Nb-chalcogenides. The abundant crossings across Fermi level in band structures demonstrate the compounds' metallicity, corroborated by Pt's and Nb's d-orbitals in PDOS calculation performed with/without SOC. In the two Nb chalcogenides, symmetry-protected nodal rings are locally gapped once SOC is included, and in Nb_2_CuS_4_ we additionally identified van-Hove-like singularities near *E*_F_. For PtN_2_, we observe the expected Kramers doublets due to time reversal symmetry together with SOC-induced avoided crossings along *K* → *M*. These results underscore robust SOC effects in reshaping low-energy dispersions among all three materials. Although a full metal–insulator transition was absent, our results affirmed their possibilities to exhibit nontrivial band topology through proper perturbations in future, as seen in comparison to previous works that successfully confirmed topological properties after appropriate tuning.^38–40^ Together, our study establishes metallic monolayer PtN_2_, Nb_2_CuS_4_, and Nb_2_CoS_4_ as promising, controllable platforms in which EPC-driven high *T*_c_ superconductivity benchmarks can be engineered by spin–orbit coupling effects to serve as potential systems capable of enabling device-relevant topological phenomena.

## Conflicts of interest

There are no conflicts to declare.

## Data Availability

All data is included in the main article.
